# An Integrated Framework to Identify Prognostic Biomarkers and Novel Therapeutic Targets in Hepatocellular Carcinoma-Based Disabilities

**DOI:** 10.3390/biology13120966

**Published:** 2024-11-24

**Authors:** Md. Okibur Rahman, Asim Das, Nazratun Naeem, Jabeen-E-Tahnim, Md. Ali Hossain, Md. Nur Alam, AKM Azad, Salem A. Alyami, Naif Alotaibi, A. S. Al-Moisheer, Mohammod Ali Moni

**Affiliations:** 1 Department of Pharmacy, Jahangirnagar University, Savar, Dhaka 1342, Bangladesh; 2 Department of Computer Science & Engineering, Jahangirnagar University, Savar, Dhaka 1342, Bangladesh; 3 Department of Computer Science & Engineering, Daffodil International University, Dhaka 1216, Bangladesh; 4 Department of Mathematics & Statistics, Imam Mohammad Ibn Saud Islamic University (IMSIU), Riyadh 13318, Saudi Arabia; 5 Artificial Intelligence and Cyber Futures Institute, Charles Sturt University, Bathurst, NSW 2795, Australia

**Keywords:** hepatocellular carcinoma, integrated bioinformatics approaches, hub genes, prognostic biomarkers, AURKA, disability research

## Abstract

Hepatocellular carcinoma (HCC) is one of the most prevalent malignant tumors globally, significantly affecting liver functions, thus necessitating the identification of biomarkers and effective therapeutics to improve HCC-based disabilities. This study aimed to identify prognostic biomarkers, signaling cascades, and candidate drugs for the treatment of HCC through integrated bioinformatics approaches such as functional enrichment analysis, survival analysis, molecular docking, and simulation. Differential expression and functional enrichment analyses revealed 176 common differentially expressed genes from two microarray datasets, GSE29721 and GSE49515, significantly involved in HCC development and progression. Topological analyses revealed 12 hub genes exhibiting elevated expression in patients with higher tumor stages and grades. Survival analyses indicated that 11 hub genes (CCNB1, AURKA, RACGAP1, CEP55, SMC4, RRM2, PRC1, CKAP2, SMC2, UHRF1, and FANCI) and three transcription factors (E2F1, CREB1, and NFYA) are strongly linked to poor patient survival. Finally, molecular docking and simulation identified seven candidate drugs with stable complexes to their target proteins: tozasertib (−9.8 kcal/mol), tamatinib (−9.6 kcal/mol), ilorasertib (−9.5 kcal/mol), hesperidin (−9.5 kcal/mol), PF−562271 (−9.3 kcal/mol), coumestrol (−8.4 kcal/mol), and clofarabine (−7.7 kcal/mol). These findings suggest that the identified hub genes and TFs could serve as valuable prognostic biomarkers and therapeutic targets for HCC-based disabilities.

## Simple Summary

Hepatocellular carcinoma (HCC) is one of the most common malignancies worldwide, with a high mortality and morbidity rate due to the absence of early clinical manifestations and appropriate biomarkers for its diagnosis. Thus, effective diagnostic and prognostic biomarkers, alongside the development of innovative treatments, stand as pivotal imperatives to improve patient outcomes more effectively. Unraveling the genetic alterations and molecular mechanisms involved in the pathogenesis of HCC may help to understand the disease and unveil potential drug targets, reshaping the treatment approach for HCC. In this context, the current study focused on identifying key differentially expressed genes from two existing datasets, GSE29721 and GSE49515, that are abnormally expressed in peripheral blood mononuclear cells and liver tissues and are also associated with worse survival of patients. Therefore, they could be used for prognostic evaluation by a non-invasive method. We believe that identified biomarkers and their pathways will provide new insights in the realm of HCC pathogenesis and treatment approaches.

## 1. Introduction

Liver cancer has emerged as the fourth-leading cause of cancer-related deaths worldwide and the sixth-most frequently diagnosed malignancy [1]. The incidence of liver cancer in men is about two times higher compared to women [2]. Several risk factors have been implicated in the pathogenesis of this disease. The most prevalent risk factors for liver cancer are chronic hepatitis B virus (HBV) [3], hepatitis C virus (HCV) [4], aflatoxin-contaminated food [5], type 2 diabetes [6], heavy alcohol consumption [7], smoking [8], and obesity [9]. There are three commonly recognized types of liver cancer, such as hepatocellular carcinoma (HCC), characterized by the malignant transformation of hepatocytes, accounting for approximately 80% of the global morbidity rate; secondly, intrahepatic cholangiocarcinoma (ICC), arising from biliary duct epithelium tissue; and finally, a rare type of hepatocellular carcinoma called combined HCC-ICC (cHCC-ICC), contributing to fewer cases worldwide [10,11,12,13]. Hepatocellular carcinoma (HCC) constitutes almost 80% of all primary liver cancer cases, posing a significant global health burden. Therefore, to ensure early diagnosis and the prediction of prognosis for patients with HCC, the identification of liver cancer biomarkers can be crucial for improving outcomes.

Due to the absence of early clinical manifestations, approximately three-fourths of primary liver cancer instances are detected and diagnosed in the advanced stage, failing to accommodate radical surgery and other treatment options, leading to poor prognosis and outcome with high recurrence and mortality rate [14]. Despite the identification of various risk factors as contributing factors to HCC, the molecular mechanism of pathogenesis has been poorly understood. Several studies have found alterations in a variety of vulnerable genes, including TP53, CTNNB1, AXIN1, cyclin D1 (CCND1), epidermal growth factor receptor (EGFR), c-myc, and Ras. These genes exhibit aberrant expression and mutations and are, therefore, implicated in the genesis and advancement of HCC [15,16,17]. Down-regulated ASPP1 and ASPP2 genes have been found to play roles in the development of HCC [18]. However, despite various studies aimed at identifying the underlying mechanisms, the current understanding is still insufficient. Therefore, further investigation is of profound importance to unveil the underlying mechanisms of HCC initiation and progression.

To date, for early-stage HCC, different interventions such as surgical resection, liver transplantation, radiofrequency ablation, and various drugs such as cisplatin and doxorubicin by transarterial chemoembolization (TACE) have been used, but the efficacy of these treatments is still insufficient due to recurrence, complex risk factors, and drug resistance [19,20]. Several targeted therapies, including sorafenib, lenvatinib, regorafenib, cabozantinib, and pembrolizumab, have demonstrated effectiveness in diverse clinical trials either alone or in combination [21]. However, the efficacy of these drugs is compromised due to the adverse effects they entail. Hence, drug repositioning or repurposing through bioinformatic tools can be a cost-effective and time-efficient approach to drug development. This approach involves exploring approved or investigational drugs that can reverse cancer-related abnormal gene expression and can be reused for HCC, thereby expanding the array of treatment alternatives [22].

Microarray technology has been used for nearly a decade to discover molecular signatures, biomarkers, and regulatory pathways in multiple malignancies, including HCC, aiding in the prediction of progression and prognosis [23]. However, the results obtained using different data processing and technological methods and small sample sizes are often contradictory, inconsistent, and insufficient [24,25]. Meanwhile, integrated bioinformatics analysis can process a large number of datasets to extract valuable biological insights, enabling the discovery of molecular mechanisms underlying pathogenesis, progression, and reliable biomarkers [26].

This study aimed to explore the core DEGs, prospective biomarkers, therapeutic targets, potential drug agents, and enriched pathways in HCC by using an integrated bioinformatics approach. Herein, we focused on screening hub genes by establishing a functional protein–protein interaction (PPI) network alongside regulatory biomarkers (i.e., TFs and miRNA) and potential therapeutic agents via drug repositioning techniques. By elucidating the enriched pathways associated with the core DEGs, the study intended to enhance our understanding of the molecular mechanisms driving HCC development and progression.

## 2. Materials and Methods

### 2.1. Differentially Expressed Gene Screening from Microarray Datasets

Microarray gene expression profile datasets were searched in the NCBI-GEO database [27]. Then, two datasets with accession numbers GSE29721 and GSE49515 were chosen from two distinguished experiments. The former comprised a total of 20 samples from 11 patients, of which 10 were micro-dissected HCC tissue and the remaining 10 were normal adjacent liver tissue derived from the Chinese National Human Genome Center in Shanghai, China [28]. GSE49515 studied 24 samples of PBMC collected from normal healthy, hepatocellular carcinoma (HCC), pancreatic, and gastric cancer patients to establish aberrant gene expression analysis using Affymetrix gene arrays in the National Cancer Centre, Singapore [29]. We used 10 healthy PBMC samples as controls and 10 PBMC samples of HCC patients as cases to find out differentially expressed genes.

Then, datasets were analyzed to compare the expression level of genes between HCC and normal tissues in the GEO2R web platform [27], where differential expression analyses were performed exercising the limma (linear models for microarray analysis) package [30]. Here, the expression matrix was log2-transformed, and Benjamini and Hochberg corrections were applied to control the false positive rate. A nominal *p*-value < 0.05 and |log2FC| > 1 were applied as the cutoff criteria, and the resultant genes that satisfied both the cutoffs were considered statistically significant DEGs. To determine common DEGs between the datasets, we used the Jvenn web application [31].

### 2.2. Gene Ontology and Pathway Enrichment Analysis of CDEGs

To figure out the molecular action and cellular action of the differentially expressed genes, functional and pathway enrichment analyses can be delineated. Therefore, the Database for Annotation, Visualization, and Integrated Discovery (DAVID; http://david.ncifcrf.gov) (accessed on 7 January 2024) [32], an open web-based biological information repository, was employed to recognize gene ontology (GO) [33] terms that include the molecular function (MF), biological process (BP), and cellular component (CC) of the CDEGs. MF indicates molecular functions of genes; BP refers to processes about the activities of multiple genes; CC annotates the place within the cell where the genes are active.

Pathway-based analysis to explore the mechanisms of complex diseases has introduced a new method called the topological-based approach that integrates both molecular and structural data of the pathway from a biological database to understand how and where genes interact with one another in a pathway [34]. Here, we considered three knowledge bases—the Kyoto Encyclopedia of Genes and Genomes (KEGG) [35], Reactome [36], and BioCarta (http://www.biocarta.com/) (accessed on 7 January 2024) in the Enrichr [37] package, a gene set enrichment analysis tool—that work in topological-based approaches to explore signaling pathways of differentially expressed genes.

GO terms and pathways were visualized in a bubble plot using the SRplot online tool [38]. For all enrichment analyses, a *p*-value < 0.05 was considered statistically significant.

### 2.3. Identification of Hub Genes

Identification of essential proteins by protein–protein interaction network topologies using high-throughput technology can be performed by analyzing the centrality of genes. Centrality measures in a PPI network provide insights into the importance of proteins within the network. Here, we exploited the STRING (Search Tool for the Retrieval of Interacting Genes) [39] database to elucidate putative PPI networks of CDEGS, and the resulting PPI networks were depicted in Cytoscape software version 3.10.1 [40], using the highest confidence score of 0.9.

The hub genes were subsequently identified using the Cytoscape plugin cytoHubba (http://apps.cytoscape.org/apps/cytohubba) (accessed on 8 Feburary 2024), which permits 11 specific ranking methods to determine hub genes from the PPI network. These methods are categorized into two types of algorithms: local-based methods and global-based methods [41]. In this study, we specifically employed two local-based methods—degree centrality (DC) and maximal clique centrality (MCC)) and two global-based methods—closeness and edge percolated component (EPC), to enhance the reliability of our findings. Afterwards, DAVID was used to carry out the KEGG and GO analyses for the hub genes.

### 2.4. Identification of Transcriptional and Post-Transcriptional Regulators

Dysregulated gene expression can be a contributing factor in several human disorders. Two major classes of gene expression regulators are transcription factors (TFs) and microRNAs (miRNAs). TFs regulate the transcriptional level of differentially expressed genes (DEGs) by binding in promoter regions, influencing the synthesis of RNA from DNA. In contrast, miRNAs control the post-transcriptional level of gene expression by binding to an area of 3′ untranslated regions of target genes, thereby negatively regulating the expression of the associated gene [42].

We used the JASPAR [43] and TarBase [44] databases to identify regulatory TFs and miRNA, respectively. We availed of the Network Analyst [45] web tool for using both the database and to visualize topological networks.

### 2.5. Validation of Hub Genes and TFs by mRNA Expression Level

UALCAN [46] is an extensive and sophisticated website housing cancer OMICS data designed to facilitate in-depth analysis of cancer transcriptomic data through the Cancer Genome Atlas (TCGA) Program. The UALCAN online database comprises 50 normal tissues and 371 HCC tissues from the TCGA database to compare mRNA expression levels. We utilized the UALCAN database to validate the expression level of the hub genes.

### 2.6. Clinicopathological Characteristics of Hub Genes

The clinicopathological characteristics mainly encompass factors such as survival analysis, tumor stage, and tumor grade. The Kaplan–Meier (KM) plotter online tool [47], which is a vast database comprising over 35 thousand samples from 21 distinct cancers, was employed to conduct the survival analysis of hub genes. This sophisticated and in-depth analysis contributes to the identification of robust prognostic biomarkers [48]. In this study, overall survival (OS) and progression-free survival (PFS) were used to find prognostic biomarkers in HCC. The UALCAN database was used to incorporate characteristic changes in expression level with tumor stages and grades.

### 2.7. Identification of Novel Candidate Drugs for HCC

In our quest to identify potential drugs that would target highly expressed hub genes, we employed drug-gene interactions through online drug repository web tools DSigDB [49] and DGIdb [50]. The DSigDB was accessed through Enrichr to predict candidate drugs. We also undertook interactions between hub genes and chemicals by the Drug–Gene Interaction Database (DGIdb), which combines a bunch of databases to predict druggable genes.

### 2.8. Molecular Docking and Simulation of Candidate Drugs

To strengthen the suggestion of repositioning drugs for HCC, we employed molecular docking and simulation techniques to scrutinize the interactions between drug agents and specific molecular targets.

The 3D crystal structure of AURKA (PDB ID: 5DT0), CCNB1 (PDB ID: 6GU2), RAGAP1 (PDB ID: 2OVJ), and RRM2 (PDB ID: 3OLJ) was collected in PDB format from the RCSB protein data bank [51], and subsequently unnecessary chains, water molecules, and co-crystalized ligands were removed using PyMOL software (Version 1.7.4). After that, the energy of the target proteins was reduced using the SwissPDB viewer (Version 4.1.0) [52]. Finally, molecular docking analyses were performed using PyRx software (version 0.8). The 3D structures of all the ligands were retrieved from the PubChem database (except cisplatin, barasertib, and paclitaxel) [53]. Amber potential was applied to detect the most stable conformer with the lowest energy using Gabedit software (version 2.5.0) [54]. Initially, all thirty energy-minimized ligands underwent docking against their respective target macromolecules. Later, using Gaussian 09 W Revision D.01, selected eight ligands were geometrically optimized employing density functional theory (DFT) [55] with the B3LYP method and 6-31G basis set to obtain more reliable binding conformation. The Discovery Studio Visualizer 2021 software was used to calculate and visualize non-bonding interactions.

Molecular dynamics simulation is widely used to examine the stability of protein-ligand complexes and to validate the results of molecular docking. In this context, normal mode analysis (NMA) dynamics simulation was conducted for the Cα atoms of the receptor proteins via the iMODS (https://imods.iqfr.csic.es/) (accessed on 13 July 2024) server [56].

## 3. Results

### 3.1. Transcriptomic Molecular Signatures Identification of HCC

**Figure 1:**
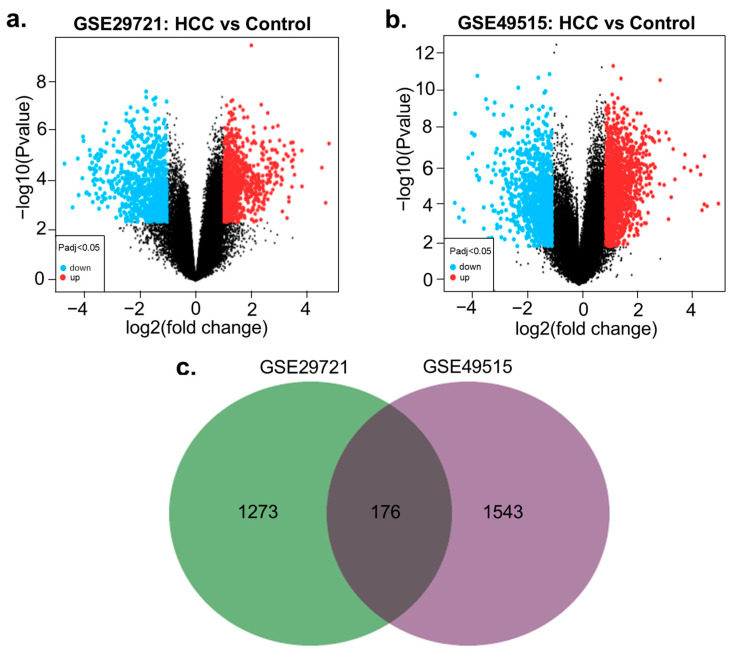
Distribution of differentially expressed genes (**a**) volcano plot for GSE29721; and (**b**) GSE49515; red dots, blue dots, and black dots indicate up-regulated genes, downregulated genes, and not significantly expressed genes in HCC compared to normal tissue, respectively. (*p* < 0.05, |log2FC| > 1) (**c**) Venn diagram showing the overlapping CDEGs between the datasets.

Differential gene expression analyses of two HCC datasets (GSE29721 and GSE49515) elucidated a total of 1449 and 1719 DEGs, respectively, after setting off the cut-off criteria. In GSE29721, analyzed between normal liver tissue and HCC tissue, 837 were up-regulated and 612 were down-regulated genes, as shown in the volcano plot (Figure 1a). In GSE49515, screened between PBMC samples from healthy patients and HCC patients, 1083 were up-regulated and 658 were down-regulated genes (Figure 1b). A total of 176 common DEGs (CDEGs) between the datasets (Table S1) were estimated and shown using a Venn diagram (Figure 1c).

The up-regulated genes and down-regulated genes exhibited an overlap of 86 and 22 differentially expressed genes, respectively (Figure S1). It was first verified whether the acquired CDEGs were connected to HCC using the DisGeNet database [57]. The top 5 diseases, ranked by overlapping genes, breast carcinoma, malignant neoplasm of breast, neoplasm metastasis, carcinogenesis, and hepatocellular carcinoma were found (*p* value < 0.05 was considered significant). Therefore, gene-disease interaction analysis reveals involvement of identified CDEGs in various carcinomas, including liver carcinoma.

### 3.2. Functional and Pathway Enrichment Analysis

**Figure 2:**
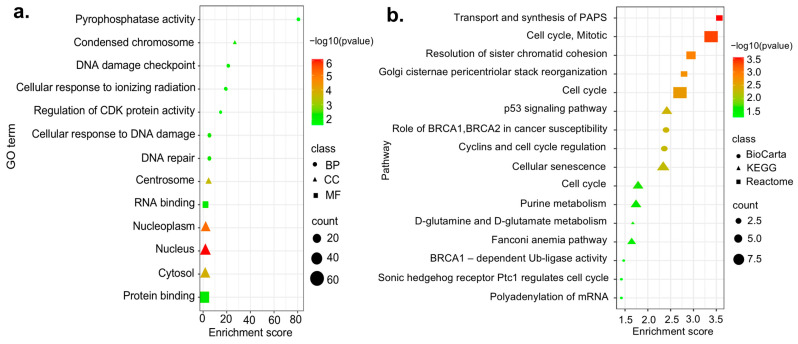
Enrichment analysis of up-regulated CDEGs (**a**) the top 5 enriched GO terms of the BP, CC, and MF categories of up-regulated DEGs; (**b**) the molecular pathways enriched by up-regulated DEGs in HCC patients.

GO enrichment analysis demonstrated that the top five biological processes (BP) of up-regulated DEGs were cellular response to DNA damage stimulus, DNA repair, DNA damage checkpoint, cellular response to ionizing radiation, and regulation of cyclin-dependent protein serine/threonine kinase activity (Figure 2a). The top five CC (cellular component) included the nucleus, nucleoplasm, cytosol, centrosome, and condensed chromosome, and the top three significantly enriched MF (molecular function) included protein binding, RNA-binding, and pyrophosphatase activity (Figure 2a).

In the pathway enrichment analysis, we used three different databases: KEGG, Reactome, and BioCarta, to reveal which pathways are altered by CDEGs. Up-regulated DEGs were significantly enriched in the 6 KEGG, 52 Reactome, and 5 BioCarta pathways. KEGG analysis showed that DEGs were primarily enriched in the p53 signaling pathway, cellular senescence, cell cycle, purine metabolism, and D-glutamine and D-glutamate metabolism (Figure 2b). The top five pathways included by Reactome were transport and synthesis of PAPS, cell cycle and mitotic, resolution of sister chromatid cohesion, Golgi cisternae pericentriolar stack reorganization, and cell cycle (Figure 2b). The roles of BRCA1, BRCA2, and ATR in cancer susceptibility, cyclins and cell cycle regulation, BRCA1-dependent Ub ligase activity, polyadenylation of mRNA, and Sonic Hedgehog receptor Ptc1 regulating cell cycle (Figure 2b) were the most enriched pathways in BioCarta. Gene ontology and pathway enrichment analysis of down-regulated genes and hub genes are presented in Figures S2 and S3, respectively.

### 3.3. Protein–Protein Interactions (PPIs) Analysis

**Figure 3:**
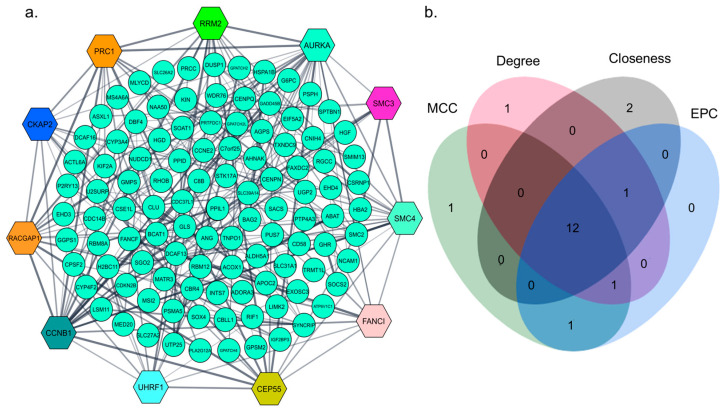
Identification of hub genes through protein–protein interaction network (**a**) PPI network of CDEGs. The nodes in hexagonal shapes represent identified hub genes; (**b**) the Venn diagram shows the intersection of 12 hub genes from four cytoHubba ranking methods.

A protein–protein interaction network predicting physical and functional associations between proteins was established using the STRING database, hiding the disconnected genes (Figure 3a). Using the four different ranking methods—MCC, degree, closeness, and EPC—of the cytoHubba algorithm, we estimated a total of 19 different top-ranked genes (Figure S4), among which 12 genes were selected as hub genes from the intersection of the topological methods (Figure 3b). These hub genes are found to play roles in multiple aspects of HCC, including tumorigenesis, progression, and metastasis (Table 1).

### 3.4. Gene Expression Regulators of CDEGs

Transcription factors (TFs) and microRNAs (miRNAs) are regulators of gene expression and biological processes, thereby influencing events associated with these processes [68,69]. We examined the interaction networks of TFs and miRNAs with CDEGs and hubs separately and selected the top 10 based on a topological parameter called degree. Among them, we identified seven TFs—FOXC1, GATA2, NFIC, YY1, E2F1, NFYA, and CREB1 (Figure S5)—and seven miRNAs—hsa-mir-1-3p, hsa-mir-124-3p, hsa-mir-16-5p, hsa-mir-34a-5p, hsa-mir-129-2-3p, hsa-mir-103a-3p, and hsa-mir-147a (Figure S6)—as mutual regulatory components for both the CDEGs and hub genes.

### 3.5. mRNA Expression of Hub Genes and TFs in Patients with HCC

**Figure 4:**
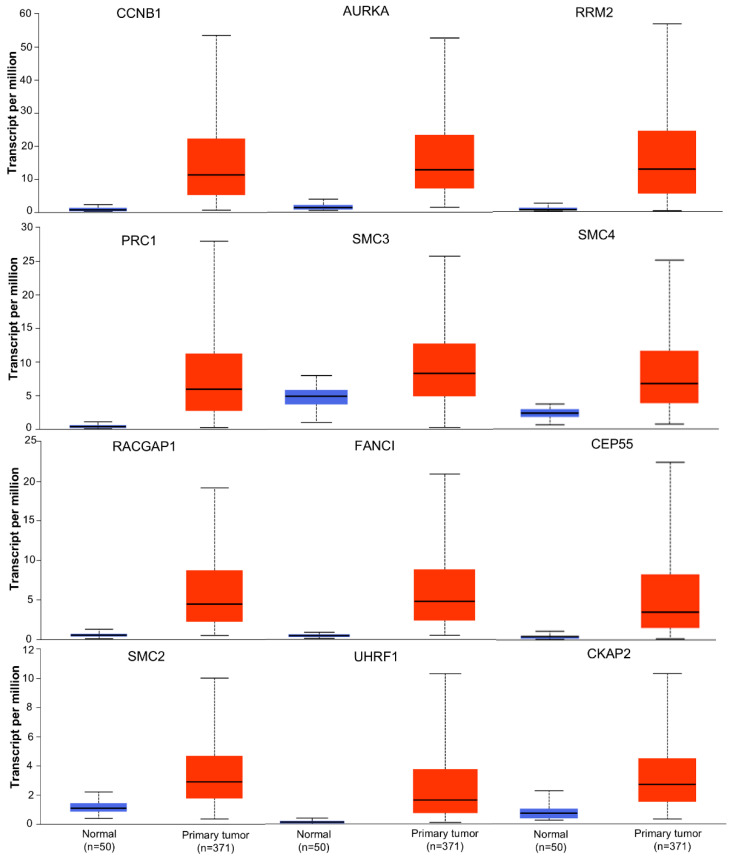
Expression levels of 12 hub genes in primary liver tumors (*n* = 371) compared to normal samples (n = 50) from TCGA data.

The mRNA expression levels of the 12 hub genes were verified from the UALCAN online database. The mRNA expression level of all twelve hub genes (CCNB1, AURKA, RACGAP1, CEP55, SMC4, RRM2, PRC1, CKAP2, SMC2, UHRF1, FANCI, and SMC3) in primary liver cancer tissues was highly up-regulated compared to normal tissues (Figure 4).

**Figure 5:**
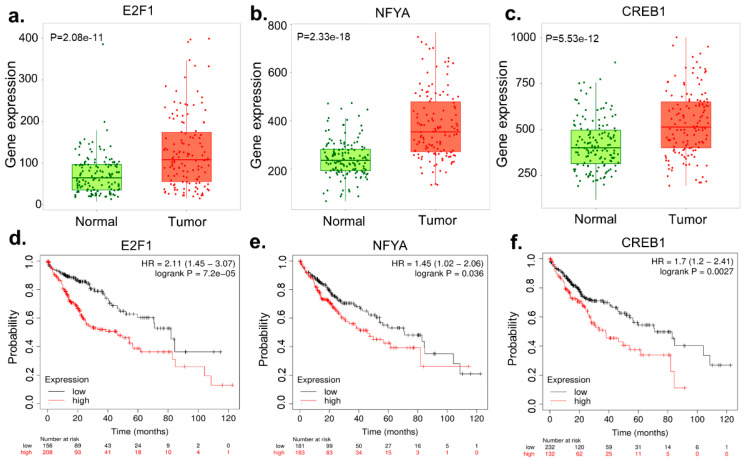
Depiction of expression patterns of three transcription factors: (**a**) E2F1, (**b**) NF-YA, and (**c**) CREB1, highlighting the upregulation of these transcription factors in liver tumors (red) compared to adjacent normal tissues (blue). Sub-figures (**d**–**f**) illustrate survival analyses for patients with high expression levels of the TFs E2F1, NF-YA, and CREB1, indicating worse overall survival in liver cancer patients with higher expression levels.

We identified seven regulatory TFs, namely FOXC1, GATA2, NFIC, YY1, E2F1, NFYA, and CREB1. Given the statistically significant result, only E2F1, NFYA, and CREB1 were overexpressed in liver tumor tissues, in contrast to their adjacent normal tissues, and were associated with worse survival for patients with liver tumors, according to the Kaplan–Meier plotter database (Figure 5).

### 3.6. Clinicopathological Characteristics of Hub Genes

**Figure 6:**
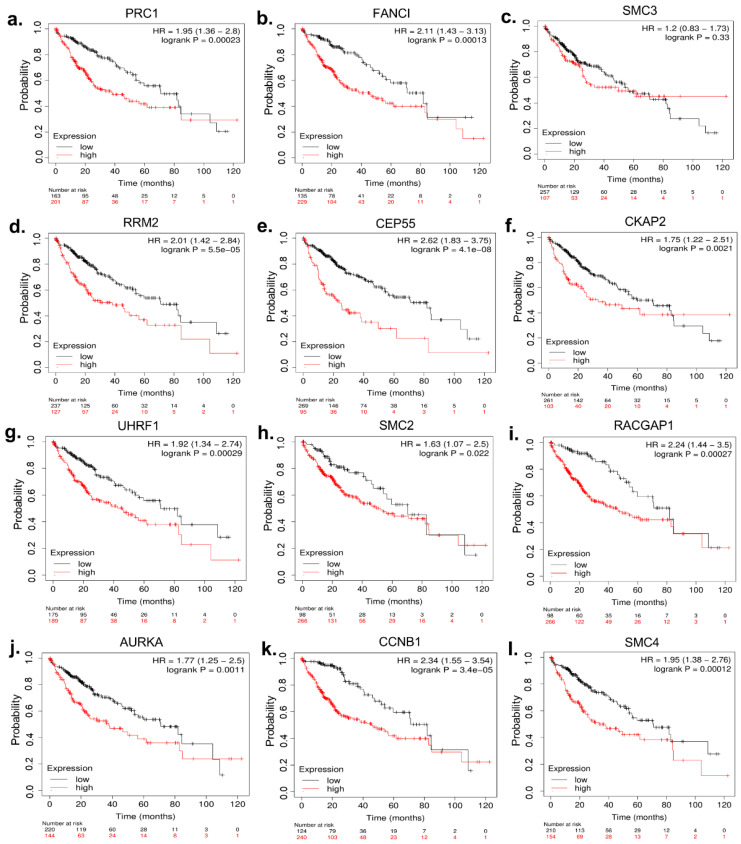
Overall survival (OS) analysis of 12 potential hub genes. *p* < 0.05 indicates a statistically significant difference in mortality between groups. Sub-figures (**a**–**l**) demonstrate that patients in the high-expression group experience significantly worse overall survival compared to the low-expression group, except for SMC3 (**c**), which is not statistically significant (log-rank *p* = 0.33). HR: hazard ratio of the two groups.

**Figure 7:**
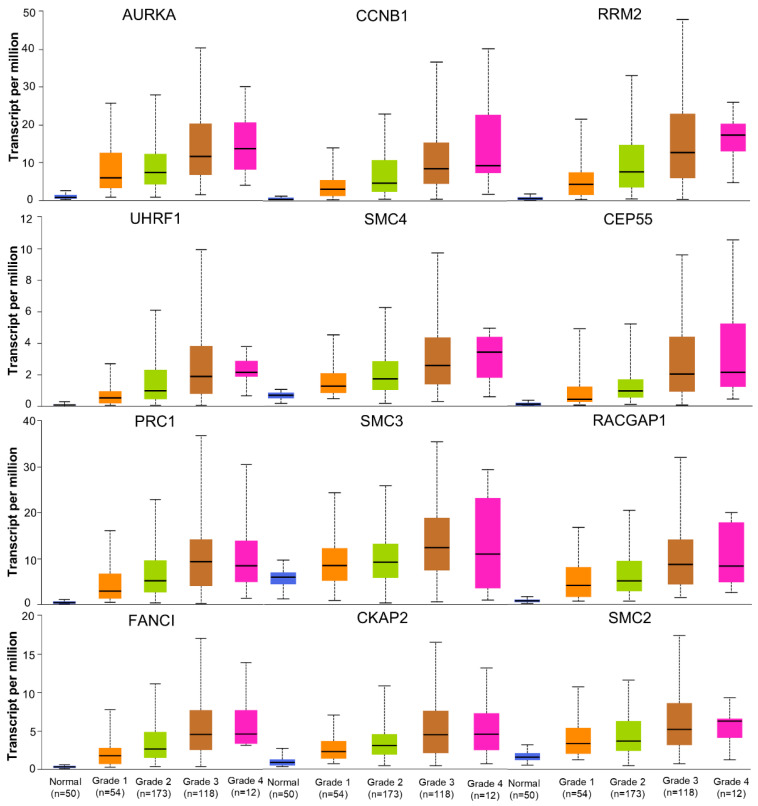
Expression of twelve hub genes in hepatocellular carcinoma patients with different tumor grades using TCGA samples. All boxplots illustrate that patients with higher tumor grades exhibit elevated gene expression in different colors.

To assess the prognostic significance of a specific gene, a cohort of 364 patient samples was divided into two groups: a low-expression cohort and a high-expression cohort, using the median expression level of the proposed biomarker as the cutoff point. A hazard ratio with a 95% confidence interval and a LogRank *p* value (<0.05 regarded as statistically significant) were used to create Kaplan–Meier survival graphs. Kaplan–Meier curves of the 12 hub genes revealed that patients with higher expression levels of these genes experienced poorer overall survival (OS) and recurrence-free survival (RFS). However, both the OS (LogRank *p* = 0.33) and RFS (LogRank *p* = 0.23) for SM3 were statistically insignificant (Figure 6 and Figure S7, respectively). According to the UALCAN database, for all the hub genes, the greater the degree of expression, the higher the grade (Figure 7) and stage (Figure S8) in HCC patients. Therefore, higher grades and stages of HCC lead to a decrease in the differentiation capability in HCC tissues.

### 3.7. Identification of Novel Candidate Drugs

To distill key insights from the analyses of gene-drug interactions, DGIdb and DSigDB were meticulously employed to identify potential therapeutics that may reverse the expression patterns of key genes. DGIdb revealed a comprehensive list of 144 drugs targeting four specific genes (CCNB1, AURKA, RACGAP1, and RRM2), with approximately 13.88% of the compounds being approved medications. DSigDB provided a total of 287 distinct drugs, gathered from different sources, applying a statistically significant *p*-value < 0.05. The drugs exhibited interactions with all the hub genes.

According to DGIdb, drugs or genes with lower interaction scores are implicated in numerous overlapping interactions and will have a higher rank in the search set [50]. Therefore, in this study, we considered the top 10 drugs with the lowest interaction scores from DGIdb, as well as the commonly identified 23 drugs between two databases, for further analyses (Figure S9).

### 3.8. Molecular Docking Analysis

Molecular docking is widely used to anticipate the binding strength and orientation of ligands within the active site of a receptor [70,71]. Here, MLN-8054 was used as the standard drug for AURKA, against which the binding affinity of other compounds was evaluated and compared [72,73]. Notably, tozasertib (−9.8 kcal/mol), tamatinib (−9.6 kcal/mol), ilorasertib (−9.5 kcal/mol), hesperidin (−9.5 kcal/mol), and PF-562271 (−9.3 kcal/mol) exhibited higher binding affinities than MLN-8054 (−9.0 kcal/mol). Clofarabine (−7.7 kcal/mol) against RRM2, and coumestrol (−8.4 kcal/mol) against CCNB1 demonstrated the highest binding energy among the drugs. Binding affinities and non-bonding interactions of ligands with the amino acid residues of the target protein are presented in Table S3.

### 3.9. Validation of the Findings Using Additional Microarray Dataset (GSE6764)

To further validate our findings, we analyzed an additional dataset (GSE6764), including HCC-diagnosed and normal liver tissue samples. We chose this dataset for two reasons: first, it uses a similar expression profiling platform to the primary liver dataset GSE29721; second, it contains samples from patients at different stages, thereby allowing us to compare our primary results with those from different disease stages. However, to maintain consistency with the previous analysis, we screened DEGs across three distinct stages in the new dataset, obtained CDEGs by comparing these results with previous findings, and conducted the remaining analyses following the same method as before.

We found 52, 76, and 114 CDEGs in the early stage, advanced stage, and very advanced stage, respectively, with two previous datasets (Table S3). Interestingly, among the previously identified 12 hub genes, 7 genes found in the early stage, i.e., AURKA, CCNB1, CKAP2, FANCI, PRC1, RACGAP1, and RRM2, were present in all three stages. CEP55, SMC4, and UHRF1 were newly identified in the advanced stage and SMC2 in the very advanced stage. However, SMC3 did not appear across any of the stages. All the hub genes were up-regulated in all stages, and their log fold change values significantly increased with the advancement in the stages, which further validated our previous findings regarding the clinicopathological characteristics of hub genes. Based on validation results, it is suggested that these seven hub genes can be used for diagnostic and prognostic purposes even at early-stage HCC. Further, we compared the functional enrichment results of the three stages with previous findings; there we found similarities to a large extent (Table S3). Therefore, these additional analysis results validate our framework by obtaining consistent results in general.

## 4. Discussion

Hepatocellular carcinoma (HCC) is the most prevalent liver tumor globally, with high rates of recurrence and metastasis. However, the early diagnostic tools and underlying pathogenesis of HCC are not fully understood, leading to a poor prognosis. Recent studies have found that aberrant gene expression and mutation are implicated in the tumorigenesis and advancement of HCC [17]. Several studies have suggested that transcriptomic alterations in peripheral blood mononuclear cells (PBMCs) are associated with multiple malignancies such as hepatocellular carcinoma [74], renal cell carcinoma, and pancreatic cancer [75]. PBMCs, which include various blood cells such as lymphocytes and monocytes, are abundant and easy to collect. PBMCs are found to contribute to tumorigenesis and poor prognosis in HCC patients by immune evasion [76]. A study found that various subsets of PBMCs, such as neutrophils, monocytes, and M1 macrophages highly infiltrating the tumor microenvironment, decrease the survival of patients [77]. Furthermore, the integration of datasets from different biological sources allows the identification of commonly dysregulated genes across tissues and the discovery of similar disease pathways, thereby strengthening our understanding of the molecular mechanisms underlying complex diseases. Therefore, we considered the study of transcriptomic changes in PBMC samples along with the liver tumor tissues of HCC patients to identify crucial biomarkers commonly expressed in both and also to enhance the validity and credibility of our findings. This approach also could potentially lead to the utilization of a less invasive method for prognostic and diagnostic evaluation.

In the current study, we utilized two GEO microarray datasets to screen differentially expressed genes between liver cancer tissues and non-cancerous tissues. To the best of our knowledge, this is the first study where PBMC samples and liver tissue of HCC patients have been used in a single study to identify crucial genes that are coordinately expressed in both samples. This study identified a total of 176 DEGs between the datasets, including 22 downregulated genes and 86 up-regulated genes. To validate our findings, we checked the expression pattern of all up- and down-regulated genes, and the results were consistent with our initial findings. Subsequently, we validated our study using a gold benchmark database such as DisGeNet.

Functional and pathway enrichment analyses were performed to explore the biological functions of DEGs and pathways associated with HCC. GO analysis of BP showed that up-regulated genes were mainly enriched in cellular response to DNA damage stimulus, DNA repair, DNA damage checkpoint, and regulation of cyclin-dependent protein serine/threonine kinase activity. Previous studies have reported that enhancement of the DNA repair pathway and mutation of the DNA damage repair pathway have been associated with tumorigenesis, development, and worse survival in hepatocellular carcinoma (HCC) [78,79]. Cyclin-dependent protein kinases (CDKs) control the regulation and expression of various components essential for cell proliferation. So, up-regulation of cyclin-dependent protein serine/threonine kinase activity may prominently implicate in the pathogenesis and progression of HCC; therefore, inhibitors of cyclin-dependent protein kinases can be promising drug targets for HCC, given their successful targeting in other types of cancer [80]. Additionally, the MF (molecular function) of up-regulated genes was significantly enriched in protein binding, RNA-binding, and pyrophosphatase activity. Studies have suggested that RNA-binding proteins (RBPs) function to form RNP (ribonucleoprotein) units or protein-protein complexes; thus, deregulated genes of RBPs are implicated in the development and progression of hepatocellular carcinoma and can serve as therapeutic targets [81,82].

KEGG analysis of up-regulated CDEGs was primarily enriched in the p53 signaling pathway, cellular senescence, cell cycle, and purine metabolism. The p53 gene governs the regulation of diverse biological processes, ranging from cell cycle arrest and apoptosis to senescence and energy metabolism to thwart tumorigenesis [83,84]. So, mutations in the tumor suppressor p53 signaling gene contribute to the proliferation, metastasis, and sensitivity to radiotherapy in HCC. The top pathways included by Reactome were cell cycle, mitotic, and resolution of sister chromatid cohesion. Prior studies have indicated that abnormalities in the cell cycle process and mitotic cell cycle are drivers in the development or advancement of HCC [85,86]. BRCA1, BRCA2, and ATR genes, along with their associated pathways, cyclins, and cell cycle regulation, as well as BRCA1-dependent Ub ligase activity, may contribute to the pathogenesis and progression of HCC, according to BioCarta [87]. The 12 up-regulated hub genes were found to be closely related to cell cycle regulation, and their dysregulated expression can lead to aberrant protein functions, ultimately resulting in uncontrolled cell proliferation, which is one of the hallmarks of cancer [88]. Functional enrichment analysis revealed some crucial processes related to cell cycle such as p53 signaling pathway (CCNB1 and RRM2), cell cycle (CCNB1 and RRM2), mitotic and G1/S transition (CCNB1 and RRM2), cell cycle checkpoints (CCNB1), G2/M transition of mitotic cell cycle (CCNB1 and AURKA), DNA repair (FANCI, UHRF1, and SMC3), and mitotic cytokinesis (RACGAP1, CKAP2, and CEP55) [Table S2]. Therefore, the cell cycle disruptions with these hub genes may contribute to the pathogenesis. Hub genes regulating processes that are related to cell cycle disruption are given in Table S2.

The study identified CCNB1, AURKA, RACGAP1, CEP55, SMC4, RRM2, PRC1, CKAP2, SMC2, UHRF1, SMC3, and FANCI as the hub genes. We found that all the hub genes were significantly overexpressed in hepatocellular carcinoma. Several studies have demonstrated overexpression of CCNB1, AURKA, CEP55, SMC2, RRM2, and PRC1, which is consistent with our study [62,63,89]. In various studies, these hub genes were suggested as prognostic biomarkers and to be involved in the development, progression, migration, and poor prognosis of HCC patients [58,59,60,61,66,67]. Moreover, high expression levels of these genes, except for SMC3, were associated with worse OS and RFS. Additionally, higher expression levels of these hub genes were found to be associated with higher tumor grade and individual tumor stage, all indicating a poor prognosis in HCC patients. Therefore, given these considerations, this study suggests that these eleven hub genes (CCNB1, AURKA, RACGAP1, CEP55, SMC4, RRM2, PRC1, CKAP2, SMC2, UHRF1, and FANCI) can be potential prognostic biomarkers and therapeutic targets in HCC.

We revealed regulatory biomolecules of identified hub genes and CDEGs with high degree values. Among the seven transcription factors (TFs), E2F1, CREB1, and NFYA were found to be overexpressed and associated with worse survival simultaneously in HCC tissue. E2F1, an important transcription factor that regulates the transcription of particular genes essential for cell cycle progression, has been found to be involved in HCC development, progression, and invasion by manipulating the expression of several genes, including MREG and DDX11 [90,91]. While inducing tumorigenesis, E2F1 activates the PI3K/AKT/mTOR pathway [92]. A previous study reported the overexpression of E2F1 in liver tumor tissues, which aligns with our findings [90]. The metastatic and proliferative characteristics of HCC cells are promoted via CREB1, driven by the HBx-CTTN coordination [93]. Furthermore, we found elevated expression of CREB1 with poor survival outcomes in HCC, which was also supported by other studies [94]. NF-YA is one of three isoforms of NF-Y, a ubiquitous trimetric transcription factor. As NF-YA regulates cell-cycle genes, any changes in expression thereby contribute to tumorigenesis [95]. NF-YA has been found to be up-regulated in various cancers, such as cervical cancer and HCC [96,97]. Additionally, it is reported that, through the MEK/ERK pathway, NF-YA promotes the proliferation and invasion of HCC cells [95].

Finally, we utilized drug repurposing strategies to find small molecular compounds capable of reversing the expression patterns of key genes. Among 33 identified drugs, this study presents seven drugs based on good binding affinity against their respective target proteins. AURKA, a member of the serine/threonine kinase family, plays a pivotal role in cell proliferation, particularly in the G2/M phase, as well as in processes such as DNA damage checkpoint recovery, centrosome maturation, and spindle formation [98,99]. AURKA has been found to be involved in tumorigenesis, affecting cell proliferation, apoptosis, and metastasis in hepatocellular carcinoma [100]. Thus, several drugs targeting AURKA have been produced and are in various phases of clinical trial. This study focused on drugs that target this gene. Tozasertib, an inhibitor of Aurora A, B, and C, is the first AKI (aurora kinase inhibitor) that has clinically been used for the treatment of CML and ALL [101]. Tozasertib exhibited the highest binding affinity against AURKA. Hesperadin, a small-molecule inhibitor with activity against Aurora A and/or B, is being developed as the first-generation AKI [102]. Ilorasertib is an aurora kinase inhibitor and has been used for phase I and phase II-advanced solid malignancies and phase I-advanced hematologic malignancies [103]. PF-562271, a FAK/Pyk2 tyrosine kinase inhibitor, has shown effectiveness in inhibiting tumor growth and recurrence when used in combination with sunitinib in rats [104]. Tamatinib is an active metabolite of the prodrug fostamatinib that has been approved for chronic immune thrombocytopenia and investigated for its effectiveness against sorafenib-resistant hepatic cell lines [105,106]. No studies have been found investigating the activity of tamatinib and ilorasertib against HCC. All the molecules targeting AURKA bind at a common site, as depicted in Figure S10. Coumestrol, a phytoestrogen, has been notified as a chemo-preventive in breast cancer [107]. However, there are currently no studies reporting coumestrol’s activity against CCNB1. Elevated expression of human ribonucleotide reductase is observed to play roles in diseases such as cancer, rendering this enzyme an appealing target for drug discovery [108,109]. According to molecular docking analysis, clofarabine was found to bind to the same site as triapine (Figure S10), with a binding affinity of −7.7 kcal/mol against RRM2 [110].

Research indicates that hydrogen bonding plays a critical role in the specificity of ligand binding, with hydrogen bonds formed within distances of 1.5 to 2.5 Å contributing to enhanced binding characteristics to different extents [111]. Significant conventional hydrogen bonds with ASP274 LYS162 have been found in almost all drugs (Figure S11). Notably, clofarabine showed hydrogen bonds with all the amino acid residues that justify strong binding interactions with proteins. Non-covalent interactions confer stability to the ligand at its target site and regulate its efficacy [112]. Deformability measures the flexibility of each amino acid residue during distortion. Distortions of all amino acids in the three proteins remained within permissible limits (Figure S12). These unequivocally signify the existence of thermodynamically favorable conformations for each of these interactions.

This study presents findings based on bioinformatics analysis; therefore, experimental validation in biological systems is essential to enhance credibility. Biomarkers should be highly specific and sensitive for use in diagnosis; thus, prospective studies with large populations are required, utilizing various techniques such as Western blot and qRT-PCR. In vitro and in vivo experiments should also be conducted to confirm the potential of repurposed drugs in clinical use. However, our study suggests potential noninvasive biomarkers that could predict the prognosis of HCC patients, as well as promising treatments for HCC.

## 5. Conclusions

The purpose of the current study was to identify prognostic biomarkers and potential therapeutic targets and reposition drugs to achieve better treatment strategies in HCC-related disabled patients by leveraging integrated bioinformatics analyses. The insights gained from this study may be of assistance in understanding the molecular mechanisms deeply driving tumorigenesis and the advancement of HCC. The hub genes and TFs uncovered in our study might hold promise as robust prognostic biomarkers and therapeutic targets, while the small molecular compounds identified may offer potential as targeted agents against HCC. However, further in vitro and in vivo validation should be undertaken to assess the safety and efficacy of the proposed drug candidates.

## Figures and Tables

**Table 1:** A summary of 12 hub proteins identified from protein–protein interaction analysis, describing their functions and affliction in HCC.

Gene	Description	Role in HCC	Ref.
CCNB1	G2/mitotic-specific cyclin-B1	Promote HCC; suggested biomarker of HCC.	[58]
SMC4	Structural maintenance of chromosomes protein 4	Contribute to the development and prognosis of tumors and prognostic markers of HCC.	[59]
RACGAP1	Rac GTPase-activating protein 1	Promotes proliferation of hepatocellular carcinoma cells; identified as a prognosis marker for early HCC detection.	[60]
AURKA	Aurora kinase A	Impelled proliferation, migration, and invasion of HCC cells.	[61]
PRC1	Protein regulator of cytokinesis 1	Identified as a prognostic marker for early HCC detection.	[60]
SMC3	Structural maintenance of chromosomes protein 3	Upregulation of the SMC3 gene has been found in HCC patients and proposed as a potential therapeutic target.	[62]
CEP55	Centrosomal protein of 55 kDa	Overexpressed and correlated with progression and poor prognosis of HCC patients; stimulates JAK2–STAT3–MMPs axis.	[63]
RRM2	Ribonucleoside-diphosphate reductase subunit M2	Suggested prognostic biomarkers and a therapeutic target for HCC.	[64]
CKAP2	Cytoskeleton-associated protein 2	Suggested novel diagnostic biomarker of HCC.	[65]
SMC2	Structural maintenance of chromosomes protein 2	Overexpressed and linked to tumorigenesis and poor prognosis in HCC.	[62]
UHRF1	E3 ubiquitin-protein ligase UHRF1	Involved in poor prognosis by promoting cell proliferation and metastasis in HCC.	[66]
FANCI	Fanconi anemia group I protein	Suggested as a diagnostic, prognostic marker, and therapeutic target.	[67]

## Data Availability

The original data presented in the study are openly available in the National Center for Biotechnology Information (NCBI) Gene Expression Omnibus (GEO) open access database (http://www.ncbi.nlm.nih.gov/geo/) (accessed on 5 Feburary 2024) with accession numbers GSE29721 [25] and GSE49515 [26].

## Supplementary Materials

The following supporting information can be downloaded at: https://www.mdpi.com/article/10.3390/biology13120966/s1, Table S1: Common differentially expressed genes (CDEGs) between GSE29721 and GSE49515; Figure S1: Up-regulated genes shared by two datasets a. Up-regulated genes b. Down-regulated genes; Figure S2: Gene ontology and pathway analysis of down regulated DEGs a. Gene ontologies of down-regulated genes, b. Enriched molecular pathways by down-regulated DEGs in HCC patients; Figure S3: GO and KEGG pathway enrichment analyses of hub genes; Table S2: Hub genes regulating processes that are related to cell cycle disruptions; Figure S4: Hub proteins identified by four different cytoHubba algorithms; a. MCC b. Degree c. Closeness d. EPC; Figure S5: Illustration of regulatory molecules in HCC. The larger the nodes in squared shape, the higher the degree of interactions and their significance. and the edges indicate the interconnection between genes and regulatory molecules a. TFs-CDEGs interaction b. TFs- hub genes interaction; Figure S6: Analysis of CDEGs/hub genes-miRNAs interaction networks reveals the post-transcriptional regulators of the CDEGs and hub genes in HCC. This figure illustrates the interactions of 12 hub genes and 176 CDEGs with their regulatory elements: a. miRNA-CDEGs interaction and b. miRNAs-hub genes interaction. Square-shaped nodes represent the miRNAs identified in this study; Figure S7: Recurrence-free survival (RFS) of 12 hub genes. *p* value < 0.05 was considered significant; Figure S8: A depiction of the expression level of hub genes in hepatocellular carcinoma patients using TCGA samples illustrate that the higher the tumor stage, the greater the expression of all hub genes. (Due to the small sample size of stage 4 (n = 6), bias in the result could arise, therefore, it was not included in the analysis); Figure S9: Summary of the identified 33 drug candidates a. 33 prospective candidate drugs with their corresponding targets according to both databases. b. Categorization of the identified candidate drugs according to advancement in therapeutic pipeline c. Drug target with their corresponding percentage of all drugs; Figure S10: Docked complex of a. AURKA (PDB ID: 5DT0) with i. PF-562271 ii. Ilorasertib iii. Hesperidin iv. Tozasertib v. Tamatinib vi. MLN-8054 b. CCNB1 (PDB ID: 6GU2) with Coumestrol c. RRM2 (PDB ID: 3OLJ) with clofarabine; Figure S11: Nonbonding interactions of a. MLN8054 b. Tozasertib c. Ilorasertib d. Tamatinib e. Coumestrol f. Clofarabine with the amino acid residues of target protein; Table S3. Nonbonding interactions of drug molecules with their targeted receptor proteins; Figure S12: Deformability of receptor proteins bounded with drug in the active site, highlighting the specific amino acid most susceptible to breakdown due to interaction with drug; a. RRM2 b. CCNB1 c. AURKA; Table S4: Comparative results between primary datasets (GSE49515 and GSE29721) and validation dataset (GSE6764)
