# Supplementary material for: An Integrated Framework to Identify Prognostic Biomarkers and Novel Therapeutic Targets in Hepatocellular Carcinoma-Based Disabilities
Source: Biology (Basel). 2024 Nov 24;13(12):966. doi: 10.3390/biology13120966 (PMC11673266; doi:10.3390/biology13120966)
Supplement: Supplementary file 1 [file biology-13-00966-s001.zip › biology-3214672-supplementary.pdf]

Supplementary file

An integrated framework to identify prognostic biomarkers and novel therapeutic targets in hepatocellular carcinoma-based disabilities

Table S1. Common differentially expressed genes (CDEGs) between GSE29721 and GSE49515

PEG10	CEP55	SLC26A2	BAG2	BTG3	ACTL6A	STRBP	RBM8A
CCNB1	CCNE2	C6orf62	GPSM2	CKAP2	CD58	EXOSC3	C10orf88
IGF2BP3	BCAT1	KLHL7	SMC2	CENPQ	STK17A	LOC284926	BLZF1
LAPTM4B	AURKA	ZNF468	GGPS1	GPATCH2	TNPO1	AGPS	DBR1
PRC1	EIF5A2	CDKN2B	ATP6V1C1	CNIH4	NUDCD1	DCAF13	STC1
B3GNT5	SMC4	DBF4	STX6	TMEM68	GLS	DCAF16	GPATCH4
SGO2	FANCI	KIF2A	PRTFDC1	DPH6	SLC35B3	RAD1	LIMK2
UHRF1	SOX4	CENPN	ZNF322	TMEM267	PRUNE1	MSI2	PTP4A3
RRM2	PSPH	RGCC	ZNF718	SMC3	C12orf49	SACS	NAA50
RACGAP1	CLEC1B	FIGNL1	HSPA1B	HINT3	SPTBN1	CSE1L	SOAT1
DIEXF	WDR76	GMPS	ASXL1	PLA2G12A	SLC39A14	DUSP1	ABAT
PRCC	GOLPH3L	INTS7	U2SURP	MIR6883	GADD45B	ALDH5A1	LOC200772
TIPRL	DLEU2	CPSF2	KIN	LOC100996412	CDC37L1	ACOX1	CLU
PPIL1	MCUR1	DESI2	TRMT1L	AVPI1	TTPAL	MLYCD	GABARAPL3
RBM33	CBLL1	MATR3	FANCF	AHNAK	KLF9	RORA	ADORA3
ZC2HC1A	RIF1	MED20	PUS7	LOC101928419	EHD3	RHOB	CBR4
EHD4	LSM11	LOC100287896	TMEM251	FAXDC2	TBXA2R	P2RY13	NAMPT
HIST1H2BJ	ZBTB9	SMIM13	ZNF765	N4BP2L1	ABHD6	CDC14B	C8B
IRF2BP2	SYNCRIP	C7orf25	GPATCH2L	MS4A6A	NDFIP2	SLC27A2	SOCS2
RBM12	TXNDC9	MIB1	MIR22	PSMA5	NCAM1	CSRNP1	SLC31A1
MBNL2	AKR7A3	HGD	HBA2	ANG	HGF	CYP4F2	GHR
UGP2	CYP3A4	APOC4-APOC2	STEAP4	G6PC	PPID	TENM1	MFAP3L

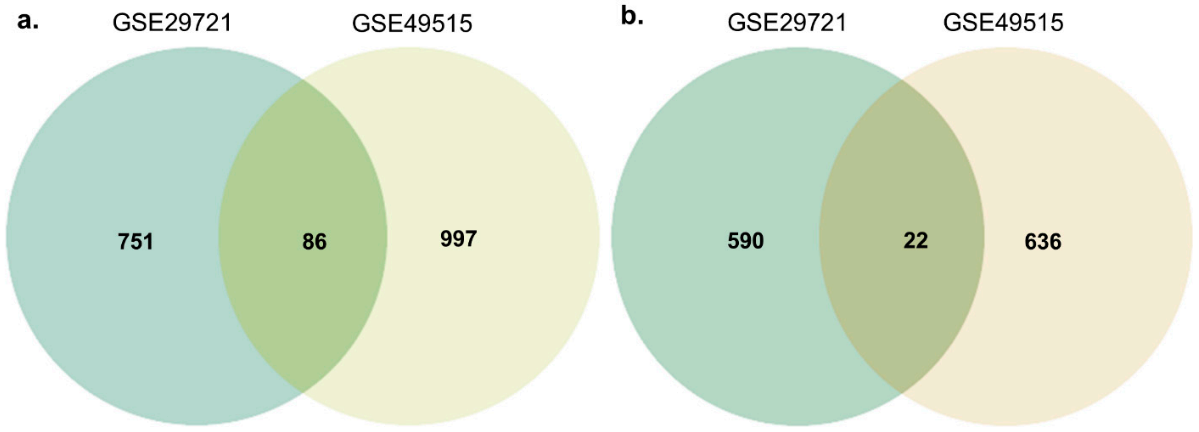

Figure S1. Up-regulated genes shared by two datasets a. Up-regulated genes b. Down-regulated genes

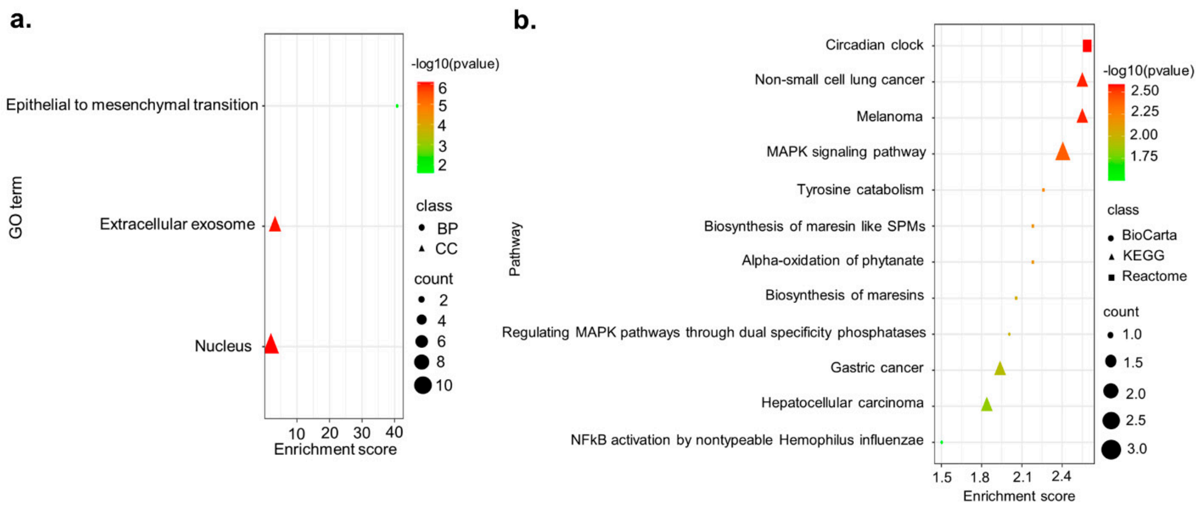

Figure S2. Gene ontology and pathway analysis of down regulated DEGs a. Gene ontologies of down-regulated genes, b. Enriched molecular pathways by down-regulated DEGs in HCC patients.

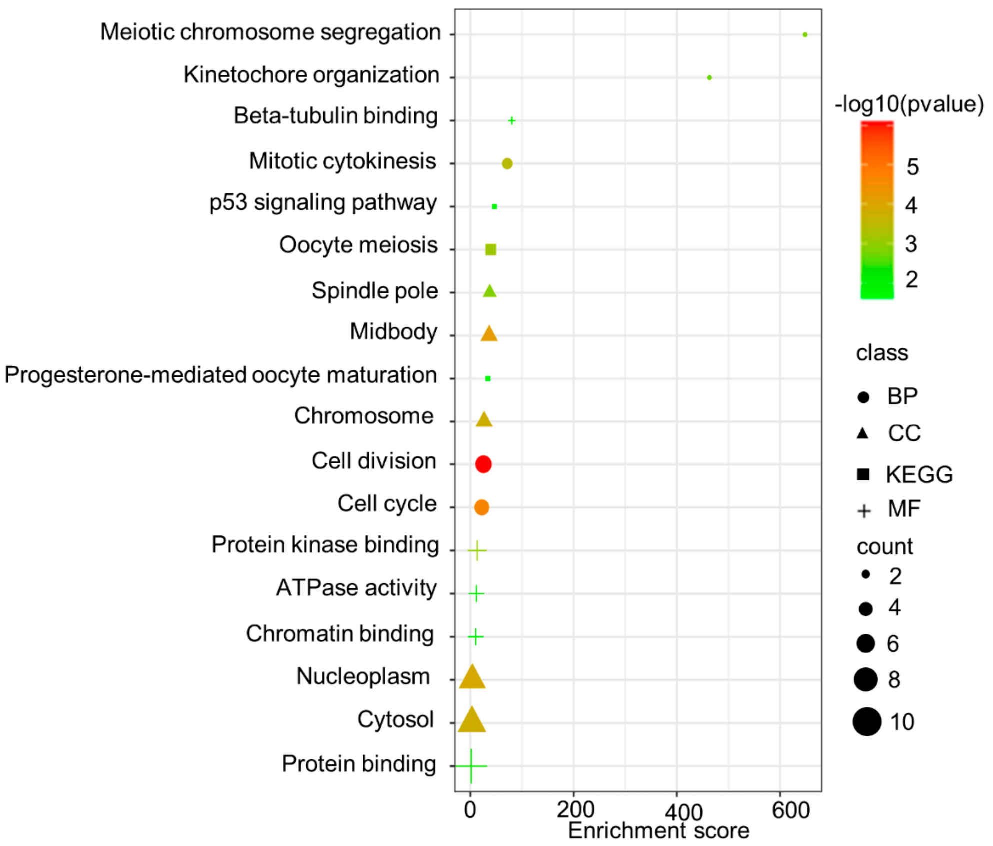

Figure S3. GO and KEGG pathway enrichment analyses of hub genes

Table S2. Hub genes regulating processes that are related to cell cycle disruptions

Term	Cell cycle related process/pathways	P-value	Gens
Biological process (BP)	cell division	1.20792E-06	CCNB1, PRC1, SMC3, SMC4, SMC2, AURKA
	cell cycle	3.66402E-05	FANCI, UHRF1, SMC4, SMC2, AURKA
	mitotic cytokinesis	0.000648862	RACGAP1, CKAP2, CEP55
	meiotic chromosome segregation	0.002824422	SMC4, SMC2
	kinetochore organization	0.003952158	SMC4, SMC2
	meiotic chromosome condensation	0.003952158	SMC4, SMC2
	positive regulation of chromosome condensation	0.005078735	SMC4, SMC2
	positive regulation of chromosome separation	0.006204154	SMC4, SMC2
	mitotic spindle midzone assembly	0.006204154	RACGAP1, PRC1
	positive regulation of chromosome segregation	0.007890112	SMC4, SMC2
	mitotic chromosome condensation	0.010694265	SMC4, SMC2
	DNA repair	0.011968551	FANCI, UHRF1, SMC3
	chromosome organization	0.017951374	SMC3, SMC4
	positive regulation of mitotic cell cycle	0.019063547	CCNB1, AURKA
	regulation of cytokinesis	0.023500797	PRC1, AURKA
	mitotic spindle organization	0.032320601	CCNB1, AURKA
	G2/M transition of mitotic cell cycle	0.032320601	CCNB1, AURKA
Molecular Function (MF)	protein kinase binding	0.0021409	CCNB1, RACGAP1, PRC1, AURKA
KEGG pathway	p53 signaling pathway	0.033811974	CCNB1, RRM2

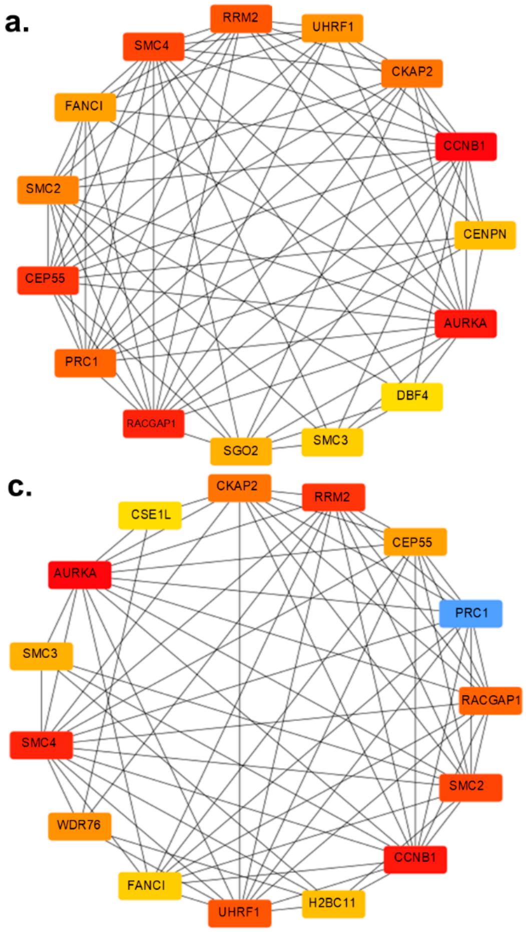

Figure S4. Hub proteins identified by four different cytoHubba algorithms; a. MCC b. Degree c. Closeness d. EPC

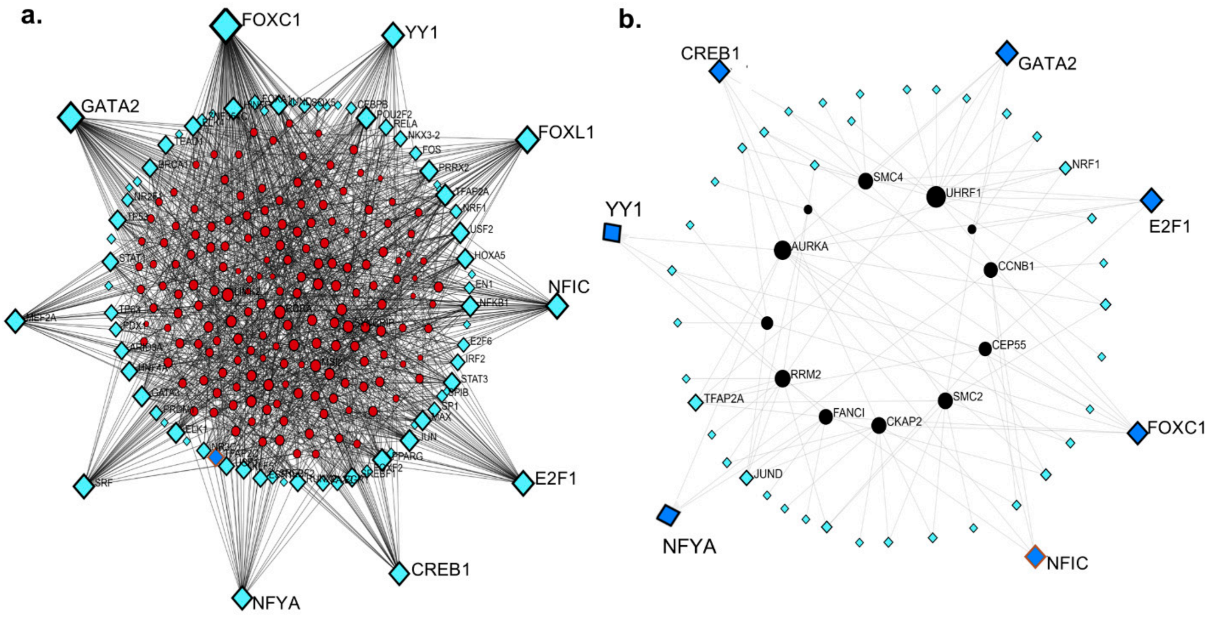

Figure S5. Illustration of regulatory molecules in HCC. The larger the nodes in squared shape, the higher the degree of interactions and their significance. and the edges indicate the interconnection between genes and regulatory molecules a. TFs-CDEGs interaction b. TFs- hub genes interaction.

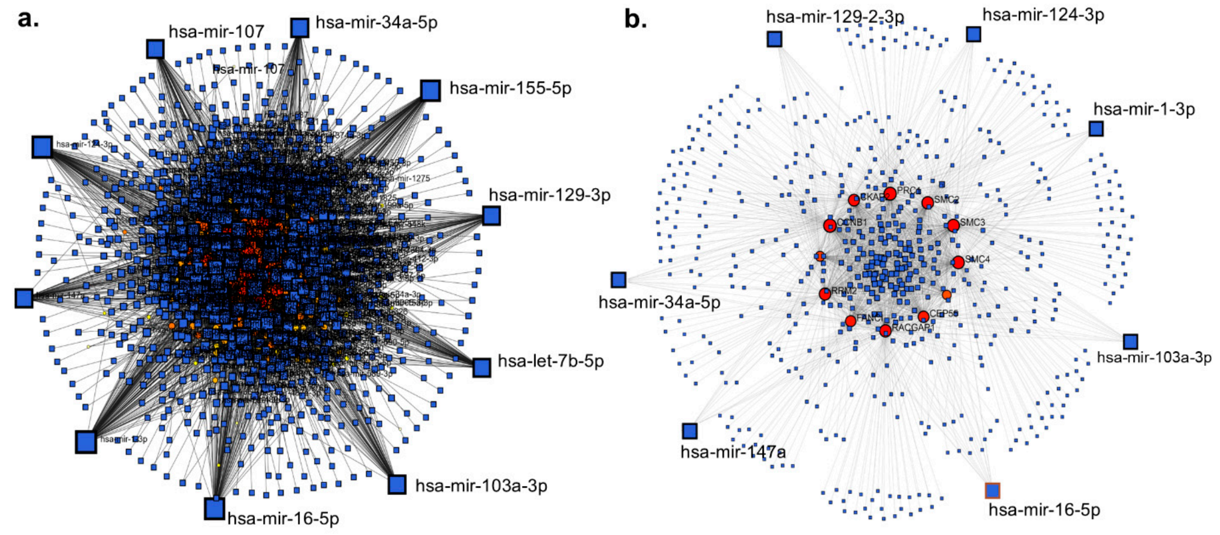

Figure S6. Analysis of CDEGs/hub genes-miRNAs interaction networks reveals the post-transcriptional regulators of the CDEGs and hub genes in HCC. This figure illustrates the interactions of 12 hub genes and 176 CDEGs with their regulatory elements: a. miRNA-CDEGs interaction and b. miRNAs-hub genes interaction. Square-shaped nodes represent the miRNAs identified in this study.

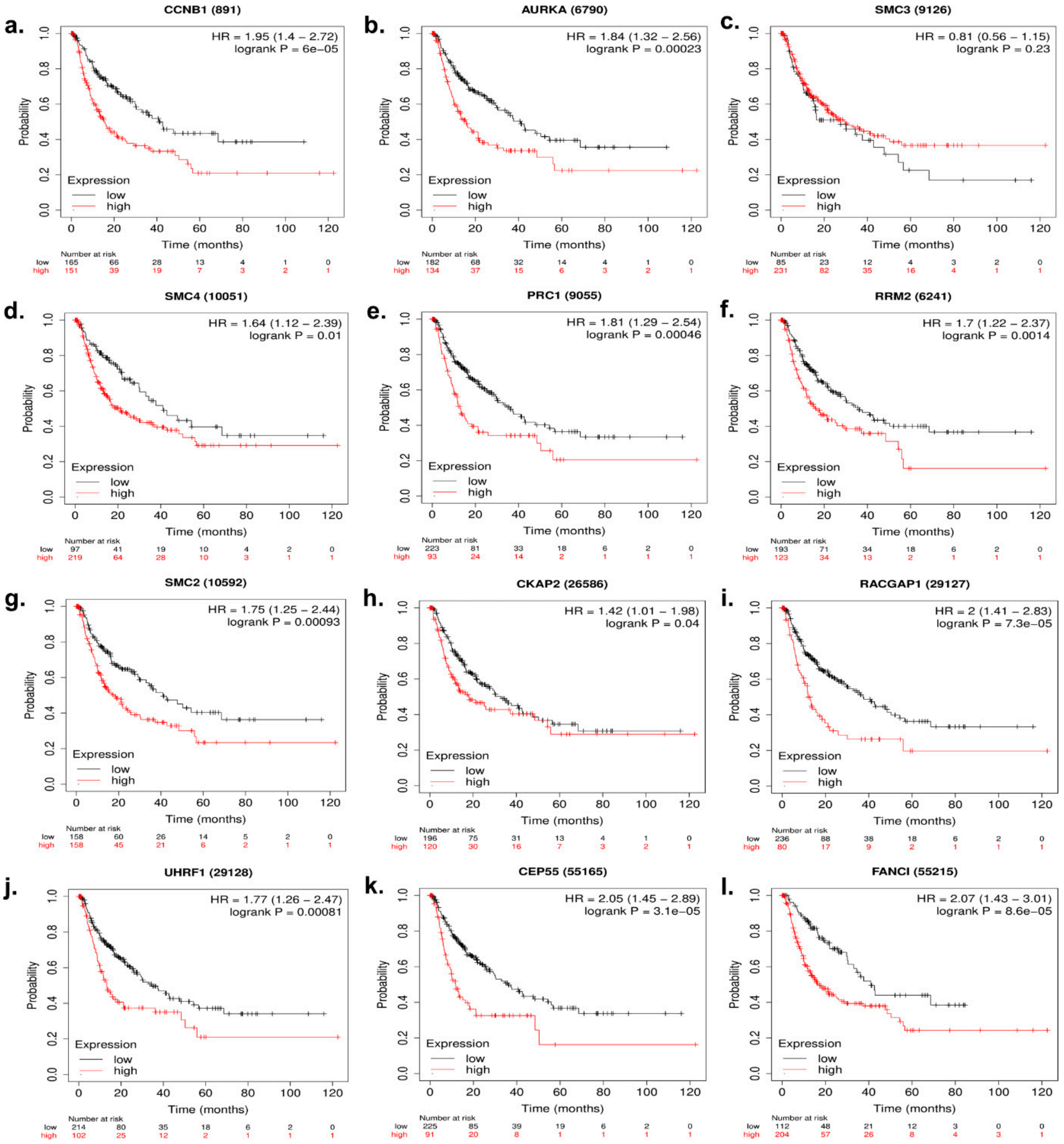

Figure S7. Recurrence-free survival (RFS) of 12 hub genes. P value <0.05 was considered significant.

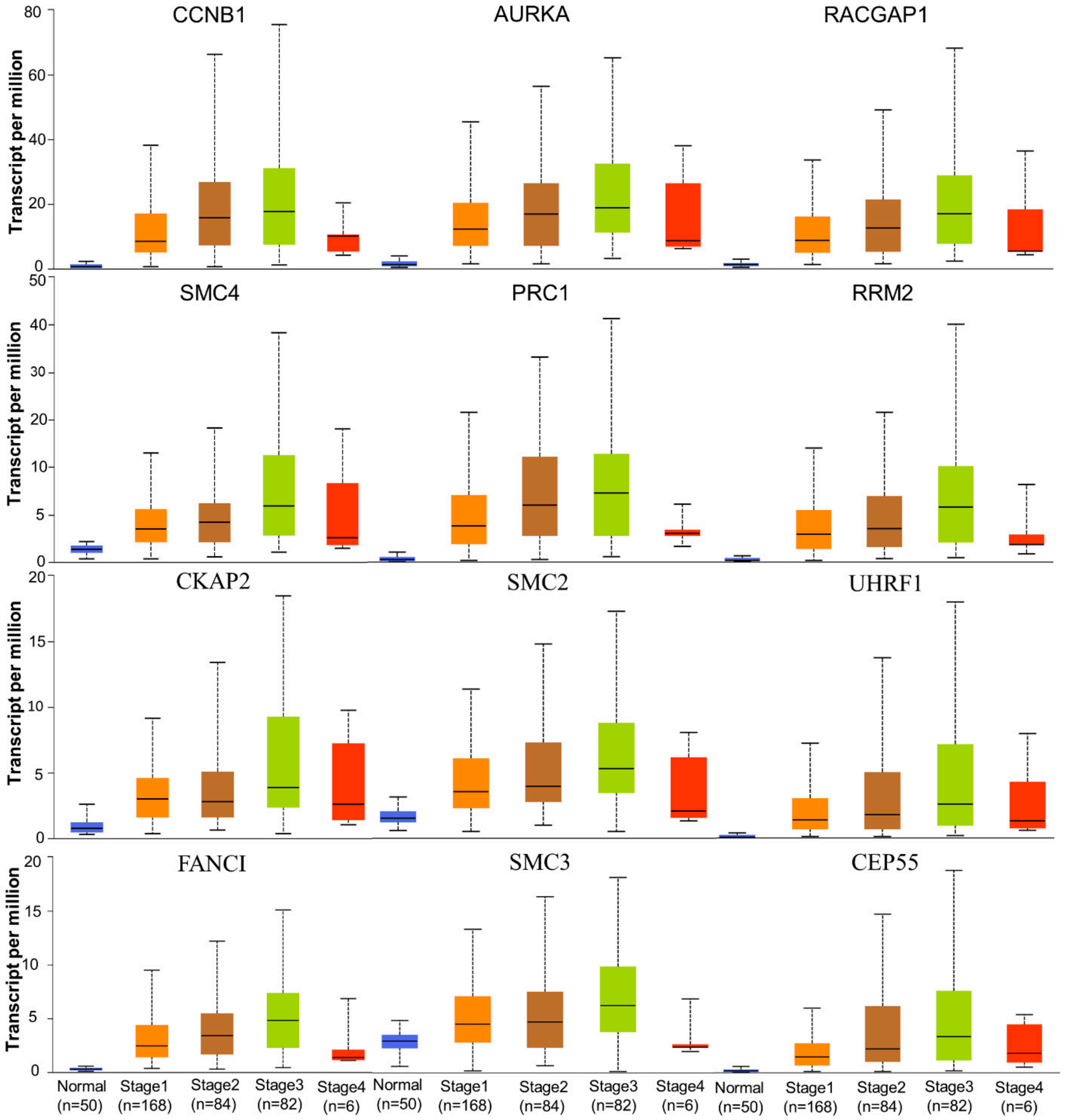

Figure S8. A depiction of the expression level of hub genes in hepatocellular carcinoma patients using TCGA samples illustrate that the higher the tumor stage, the greater the expression of all hub genes. (Due to the small sample size of stage 4 (n=6), bias in the result could arise, therefore, it was not included in the analysis).

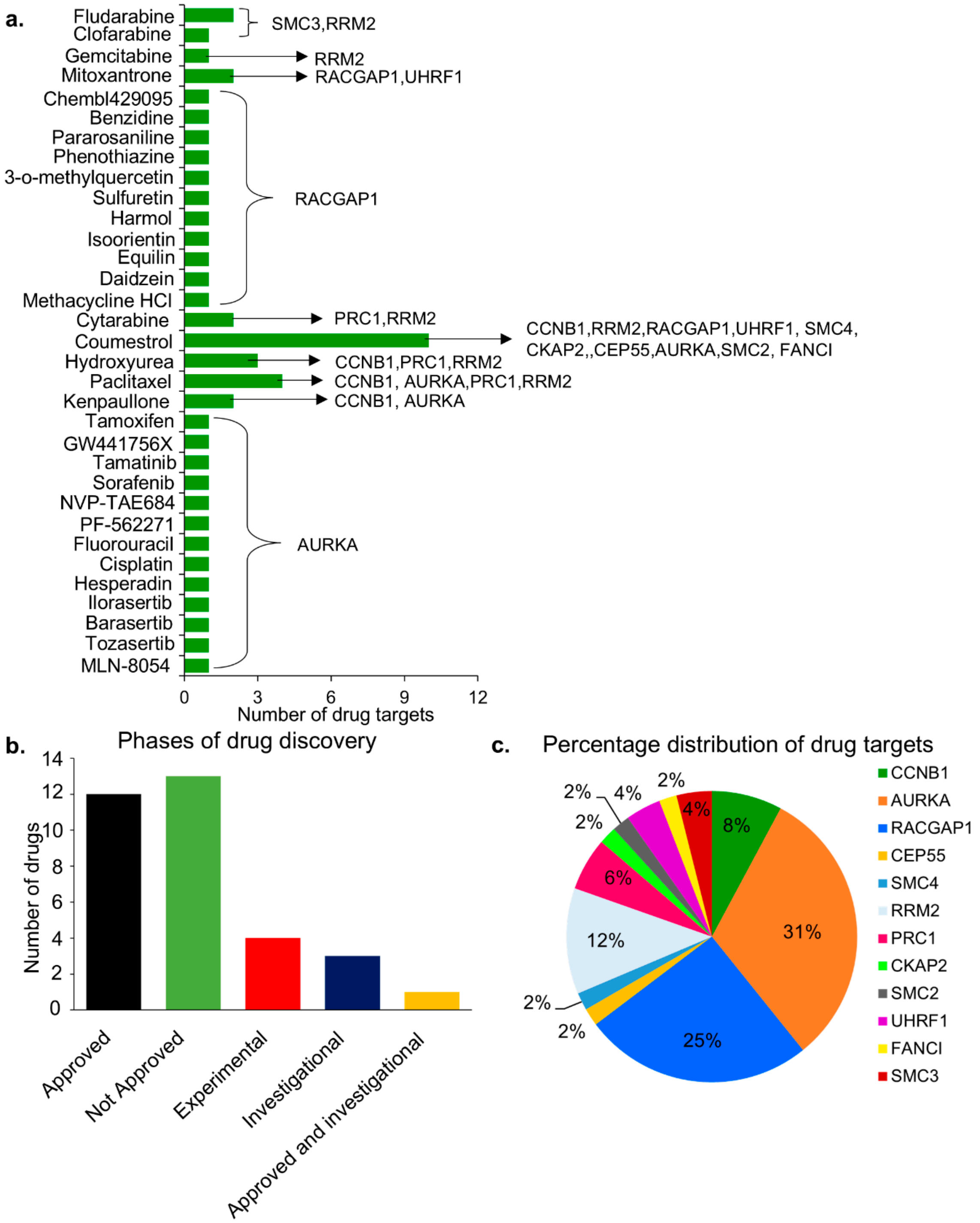

Figure S9. Summary of the identified 33 drug candidates a. 33 prospective candidate drugs with their corresponding targets according to both databases. b. Categorization of the identified candidate drugs according to advancement in therapeutic pipeline c. Drug target with their corresponding percentage of all drugs.

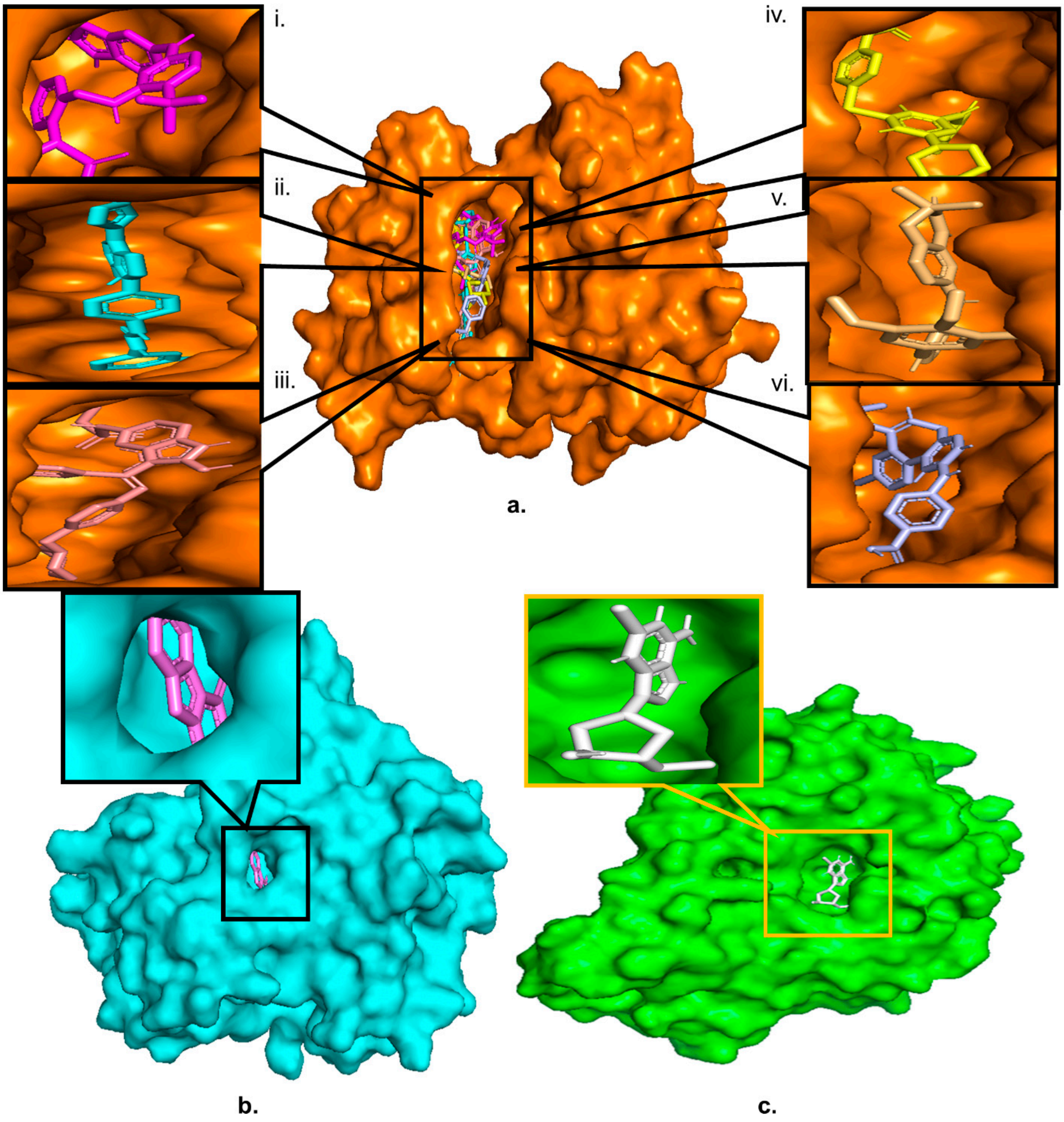

Figure S10. Docked complex of a. AURKA (PDB ID: 5DT0) with i. PF-562271 ii. Ilorasertib iii. Hesperidin iv. Tozasertib v. Tamatinib vi. MLN-8054 b. CCNB1 (PDB ID: 6GU2) with Coumestrol c. RRM2 (PDB ID: 3OLJ) with clofarabine.

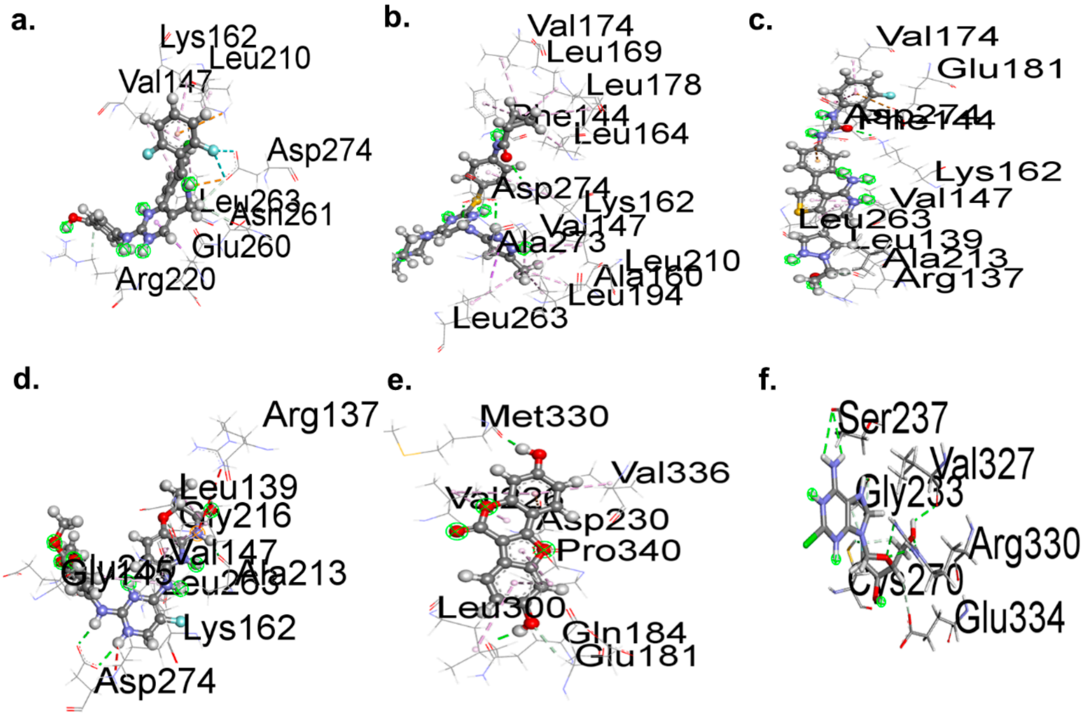

Figure S11. Nonbonding interactions of a. MLN8054 b. Tozasertib c. Ilorasertib d. Tamatinib e. Coumestrol f. Clofarabine with the amino acid residues of target protein.

Table S3. Nonbonding interactions of drug molecules with their targeted receptor proteins

Name	Binding affinity (kcal/mol)		Residues in contact	Interaction's type	Bond distance (Å)
	Before optimization	After optimization			
MLN-8054	-9.0	-9.0	ASP274	Attractive Charge	3.52
			ARG220	Carbon Hydrogen Bond	2.52
			ASN261	Carbon Hydrogen Bond	2.71
			ASP274	Halogen (Fluorine)	3.38
			ASP274	Halogen (Fluorine)	3.19
			LYS162	Pi-Cation	4.51
			GLU260	Pi-Sigma	2.82
			VAL147	Alkyl	4.30
			LEU210	Alkyl	4.83
			LEU263	Alkyl	5.37
			LYS162	Pi-Alkyl	4.55
			VAL147	Pi-Alkyl	5.22
Ilorasertib	-9.5	-9.3	LYS162	CHB*	2.19
			LEU139	Carbon Hydrogen Bond	2.55
			ALA213	Carbon Hydrogen Bond	2.68
			GLU181	Pi-Anion	4.50
			ASP274	Pi-Anion	3.61
			PHE144	Pi-Pi T-shape	5.07
			VAL147	Pi-Alkyl	4.85
			VAL147	Pi-Alkyl	4.16
			LEU263	Pi-Alkyl	4.86
			VAL174	Pi-Alkyl	4.94
Tamatinib	-9.6	-9.3	ALA213	CHB	2.66
			ASP274	CHB	2.13
			ASP274	CHB	2.16
			GLY216	Carbon Hydrogen Bond	2.47
			GLU260	Carbon Hydrogen Bond	2.93
			GLY145	Carbon Hydrogen Bond	2.51
			LEU139	Alkyl	3.90
			VAL147	Alkyl	4.80
			LEU139	Pi-Alkyl	4.28
Coumestrol	-8.5	-7.5	GLN184	CHB	1.91
			MET330	CHB	1.81
			GLU181	Carbon Hydrogen Bond	2.64
			ASP230	Pi-Anion	3.88
			PRO340	Pi-Alkyl	4.46
			VAL226	Pi-Alkyl	4.92
			LEU300	Pi-Alkyl	5.34
			PRO340	Pi-Alkyl	3.83
			VAL226	Pi-Alkyl	5.16

			VAL336	Pi-Alkyl	5.20
Hesperadin	-9.5	-9.3	ASP274	Attractive Charge	2.95
			LYS162	CHB	2.07
			GLU260	CHB	2.88
			ASP274	Pi-Anion	3.68
			LEU139	Alkyl	4.85
			VAL147	Alkyl	5.22
			ALA160	Alkyl	4.15
			ALA213	Alkyl	4.78
			LEU263	Alkyl	4.36
			VAL147	Pi-Alkyl	5.47
			VAL147	Pi-Alkyl	4.91
PF-562271	-9.3	-8.4	GLU181	CHB	2.13
			LYS162	Carbon Hydrogen Bond	2.46
			GLY145	Carbon Hydrogen Bond	2.89
			GLU260	Halogen (Fluorine)	3.19
Tozasertib	-9.8	-9.1	LYS162	CHB	2.19
			ASP274	CHB	2.21
			ASP274	CHB	2.25
			LEU263	Pi-Sigma	2.93
			ALA160	Alkyl	4.10
			LEU164	Alkyl	4.98
			LEU169	Alkyl	5.45
			VAL174	Alkyl	5.01
			LEU178	Alkyl	4.57
			LEU194	Alkyl	4.39
			LEU210	Alkyl	5.33
			LEU260	Alkyl	4.60
			PHE144<br>LYS162	Alkyl	4.91<br>[illegible]
			VAL174	Pi-Alkyl	5.23
			LEU210	Pi-Alkyl	4.89
			ALA273	Pi-Alkyl	5.46
Clofarabine	-7.7	-8.1	CYS270	CHB; Halogen (Fluorine)	2.15
			ARG330	CHB	2.39
			ARG330	CHB	2.17
			ARG330	CHB	2.65
			GLY233	CHB	2.90
			SER237	CHB	2.74
			SER237	CHB	3.03
			VAL237	CHB	2.14
			GLY233	Carbon Hydrogen Bond; Halogen (Fluorine)	2.75

*CHB= Conventional Hydrogen Bond.

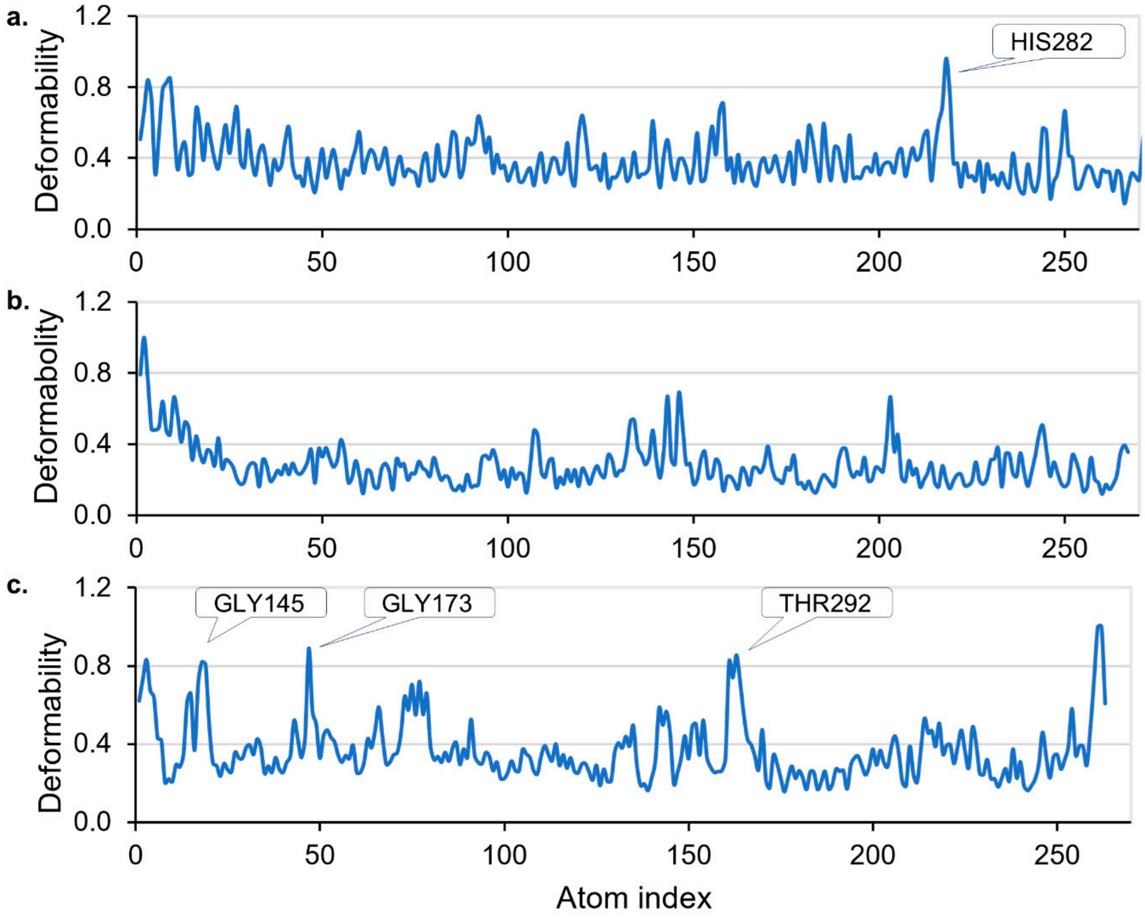

Figure S12. Deformability of receptor proteins bounded with drug in the active site, highlighting the specific amino acid most susceptible to breakdown due to interaction with drug; a. RRM2 b. CCNB1 c. AURKA.

Table S4. Comparative results between primary datasets (GSE49515 and GSE29721) and validation dataset (GSE6764)

Parameter	Primary two datasets	Validation dataset (GSE6764)		
		Early-stage HCC	Advanced HCC	Very advanced HCC
DEGs*	-	1569	2162	3470
Common DEGs	176	57	76	114
Hub genes	12	13	14	14
Similar hub genes	-	7	10	11
Common KEGG Pathway	-	p53 signaling pathway, Cellular senescence, Cell cycle, Purine metabolism	Same as our previous findings	Same as our previous findings
Common Reactome Pathways	-	Cell Cycle, Mitotic, Resolution of Sister Chromatid Cohesion	Cell Cycle and Mitotic	Cell Cycle and Mitotic, and Resolution of Sister Chromatid Cohesion
Common BioCarta Pathways	-	Cyclins and Cell Cycle Regulation, Sonic Hedgehog Receptor Ptc1 Regulates cell cycle	Cyclins and cell cycle regulation, BRCA1-dependent Ub-ligase activity, and Sonic Hedgehog Receptor Ptc1 regulating cell cycle	BRCA1, BRCA2, and ATR in cancer susceptibility, cyclins and cell cycle regulation, BRCA1-dependent Ub-ligase activity, and Sonic Hedgehog Receptor Ptc1 regulating cell cycle

*DEGs= differentially expressed genes
